# Open laboratory notebooks: good for science, good for society, good for scientists

**DOI:** 10.12688/f1000research.17710.2

**Published:** 2019-04-02

**Authors:** Matthieu Schapira, The Open Lab Notebook Consortium, Rachel J. Harding

**Affiliations:** 1 Structural Genomics Consortium, University of Toronto, Toronto, ON, M5G 1L7, Canada; 2 Department of Pharmacology and Toxicology, University of Toronto, Toronto, ON, M5G 1L7, Canada

**Keywords:** open lab notebooks, open science, peer-review, preprints, publishing, science communication

## Abstract

The fundamental goal of the growing open science movement is to increase the efficiency of the global scientific community and accelerate progress and discoveries for the common good. Central to this principle is the rapid disclosure of research outputs in open-access peer-reviewed journals and on pre-print servers. The next bold step in this direction is open laboratory notebooks, where research scientists share their research — including detailed protocols, negative and positive results — online and in near-real-time to synergize with their peers. Here, we highlight the benefits of open lab notebooks to science, society and scientists, and discuss the challenges that this nascent movement is facing. We also present the implementation and progress of our own initiative at openlabnotebooks.org, with more than 20 active contributors after one year of operation.

## Introduction

The function of the scientific peer-reviewed system is to provide greater confidence that published research is scientifically sound. This system is widely accepted as the best available, although imperfect (as peer reviewers may miss technical flaws or be biased) ^1^, to guide the global scientific community towards progress. Peer-reviewed publishing is also used by research scientists, funders and institutions as a mechanism to claim ownership of their discoveries. As a result, the community widely believes that findings should be kept secret until they are published in a peer-reviewed journal. This tradition of secrecy, which protects the scientist as opposed to the science, has been transmitted from mentor to trainee for centuries (Galileo kept his discoveries to himself until they were published). In the life sciences, this belief can reach near-mystical levels ^2^, and can be compounded by constraints associated with patent protection procedures or the absence of clear mechanism to make one’s data publicly available. The peer-review and publication process grew in an era where communication was largely in paper format. Today, in the age of instant communication, one would imagine there should be more efficient ways to operate.

## Open lab notebooks: good for science and society

We believe that open laboratory notebooks, where research scientists record their work online and in near-real time, are an efficient way to disseminate data before it is published in peer-reviewed journals, and has several advantages over the traditional “release after publication” system ^3^. First, making the data accessible within weeks rather than keeping it hidden for years means that others will be able to build upon the research, and avoid spending time and resources on redundant experiments ^4^. Second, open lab notebooks should include detailed protocols that can be reproduced, which is often not the case in peer-reviewed publications ^5, 6^. Third, negative data, which are almost never disclosed in the current publishing system but are provided in open lab notebooks, can sometime provide important insight ^7, 8^. Fourth, open lab notebooks offer a space for anyone to comment on experimental records. This allows experts to provide insight, but also to flag technically unsound experiments, thereby reducing the potential for flawed science to appear in peer-reviewed journals and in pre-print media. Open lab notebooks can therefore help save time, resources, and knowledge. If adopted by many, they should lead to a more synergistic way to do science and to more efficient use of public funds.

## Good for scientists

Many believe that the chances of getting scooped before one publishes their work in a peer-reviewed journal increase when openly sharing their work online ^9^. We argue that open lab notebooks have compensating advantages that are good for scientists. To succeed in academia, one must get funding, assert primacy over discoveries, be known in a field of research and be able to present work and ideas clearly and convincingly. Open lab notebooks can help in all aspects.

First, funding agencies are seeing the open science movement as a long lasting and far-reaching shift for the best, and are increasingly supportive of efforts to embrace open science principles. For instance, the symposium set to launch openlabnotebooks.org was entirely sponsored by the Wellcome Trust and the Canadian Institute of Health Research, and senior representatives from the Gates Foundation and the Chan-Zuckerberg Initiative were also in attendance ( https://www.thesgc.org/open-lab-notebooks-2018). The NIH’s National Institute on Aging dedicated an entire session to open science at their 2018 Alzheimer’s research summit ( https://www.nia.nih.gov/research/nih-ad-summit-2018-program-agenda), as did the 2018 Enroll-HD congress of the CHDI Huntington’s Disease Foundation ( https://www.enroll-hd.org/enroll-hd-congress-2018/). The Wellcome Trust has recently launched the Wellcome Open Research publishing platform and Open Research Fund. Our personal observations seem to indicate that grant applications highlighting the use of open lab notebooks are being viewed positively. For example, Huntington’s disease (HD) research funders such as the CHDI Foundation, the Huntington Society of Canada and the Huntington Society of America, have all generously funded studies of HD biochemistry at the SGC Toronto.

Second, results in open lab notebook are date-stamped, thus claiming temporal priority of the data. Indeed, public repositories such as Zenodo ^10^ add a date-stamp to depositions, and assign a citable DOI to open lab notebook records (detailed below): once a record has been published, it can no longer be modified, but revised versions can be appended if necessary.

Third, early career scientists can use their open notebooks to connect with their peers and with experts in the field, start new collaborations and build their own network. Fourth, the use of open lab notebooks provides opportunity to present work clearly and concisely to both experts and non-experts. This is an important skill to master in order to write convincing grant applications. Fifth, junior scientists will also find their open lab notebook a good medium to showcase their technical skills and scientific insight, and may find it useful to add a link in their resume when applying for their next position. Finally, many will find a personal satisfaction in embracing open science and FAIR data principles ^11^.

## Implementation of an open lab notebook platform

Open lab notebooks have been pioneered and championed by a number of practitioners but remain a niche activity in the scientific community. Jean-Claude Bradley first coined the term “open notebook science” in 2006 and his definition of this method of scholarly communication have laid the foundations for our own efforts ^12^. In addition to the notebooks of individual researchers following Bradley’s template, open notebook examples now include collective efforts from the Open Lab Notebook Network ( http://onsnetwork.org) and Open Source Malaria ( http://opensourcemalaria.org). However, the open lab notebook community remains small, the practice is not consistently defined or implemented and the impact of these efforts in the field have not been systematically evaluated.

**Figure 1.**
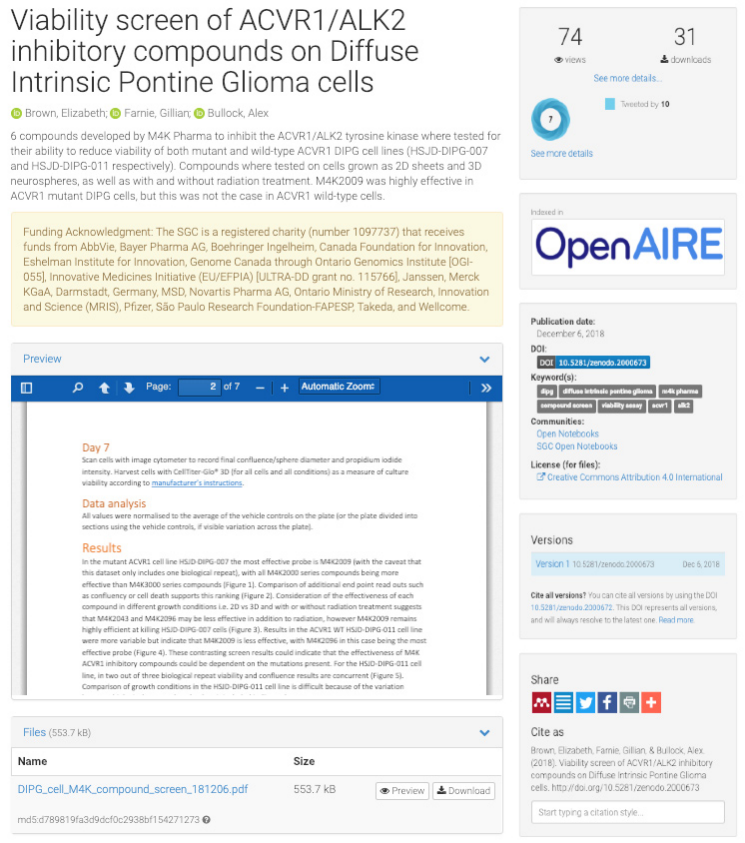
Detailed experimental records including protocols, positive and negative data are posted on Zenodo. A citable DOI is automatically generated (right-middle panel), and the number of visits and downloads provided (top right, numbers as of December 2018).

**Figure 2.**
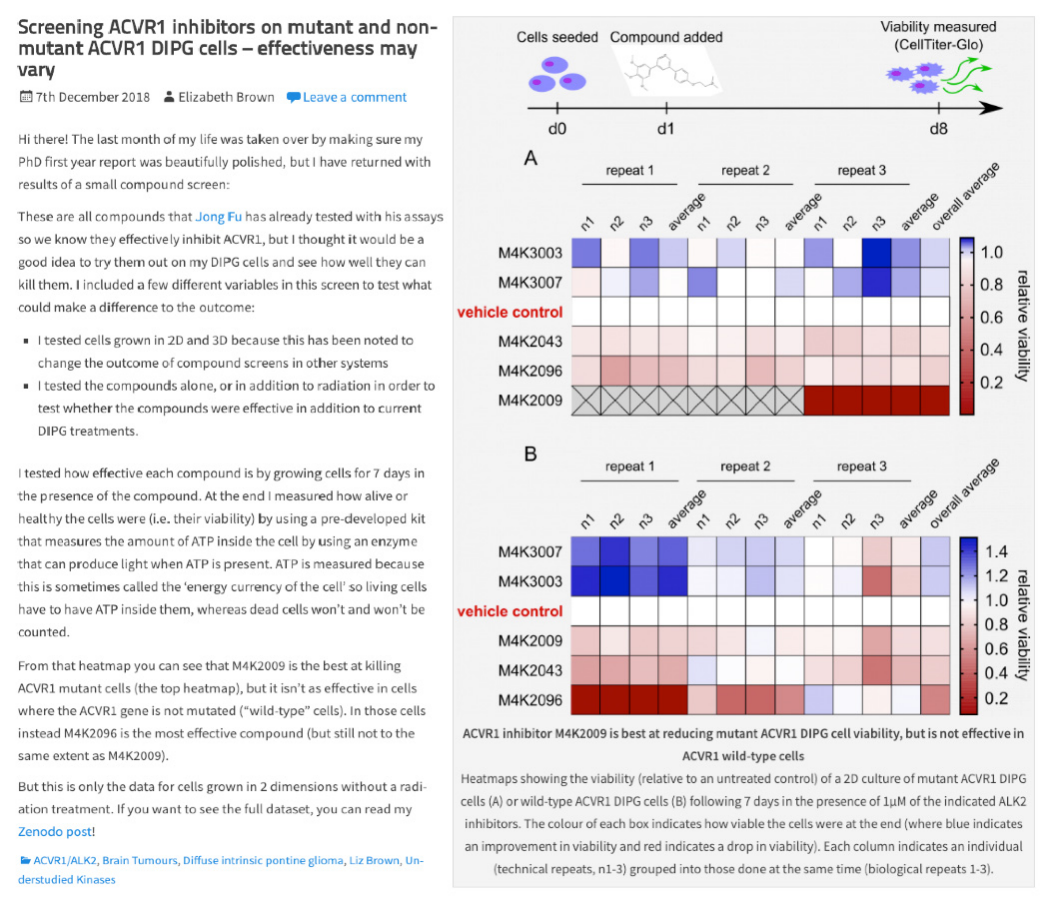
A blog explains in language accessible to non-scientists (such as patient groups) the rationale and take-home message of the experiment.

Following our prediction that open lab notebooks should be good for science and good for scientists, and after a 2-year pilot study where Rachel Harding, a post-doctoral fellow at the Structural Genomics Consortium (SGC) shared her work on Huntington’s disease at labscribbles.com ^13^, we launched openlabnotebooks.org in January 2018, where 12 scientists from the SGC started reporting their work live, online ^14, 15^. Each post is composed of two documents. (1) A detailed and rigorous experimental record, including all data and protocols, which experts can evaluate, comment on or build upon ( Figure 1); (2) a blog, aimed at the non-specialist that explains in simple terms the motivation and rational for the experiment, summarizes results – positive and negative – and outlines next steps ( Figure 2). The blogs, posted at openlabnotebooks.org, are managed by a webserver downloaded from wordpress.org (the open-source online system LabTrove would be a valid alternative ^16^), archived weekly to GitHub (repository https://github.com/thesgc/static-openlabnotebooks), quarterly to archive.org ( https://wayback.archive-it.org/6473/*/https:/opennotebook.thesgc.org/), and link to the experimental records, which are deposited at Zenodo (zenodo.org), but can also be made available from other public repositories, such as GitHub (github.com) or Figshare (figshare.com). While the experimental details posted at Zenodo are important scientifically, the blog written in layman’s term can be used to engage with scientists that may have a complementary set of expertise for future collaborations as well as other stakeholders in the research process, including patient groups, a dimension that most in academia are missing.

The Zenodo repository enables sharing research outputs from across all fields of research, creation and curation of complete digital repositories, flexible licensing with controlled degree of openness and safe storage of the data for the future in the same cloud infrastructure as CERN’s own LHC research data. Open laboratory notebooks need to guarantee that the data will remain accessible, in order to avoid the fate suffered by the pioneer open notebook of Jean-Claude Bradley, which is still accessible while its associated raw data wiki is not. Zenodo is strongly committed to preserving the data it archives. CERN has existed since 1954 and has an experimental program defined for the next 20+ years. Each file has two replicas located on different disk servers. In the highly unlikely event that Zenodo closes operations, they guarantee migration of all content to other suitable repositories, and since all uploads have DOIs, citations and links to Zenodo resources (including data) will not be affected.

**Figure 3.**
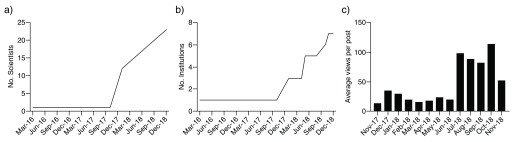
Information on openlabnotebooks.org. The Number of ( **a**) scientists and ( **b**) institutions actively contributing to openlabnotebooks.org. ( **c**) The average number of unique visits for each experimental record.

The ultimate goal of this open lab notebook initiative is not only to increase the impact of our work but also, along with precursors in the field such as Open Source Malaria ( http://opensourcemalaria.org/) and other isolated open lab notebook efforts, to inspire others to follow, and contribute to the creation of a new open science movement in the life sciences. While it is too early to judge the success of this initiative, the number of contributing scientists and institutions is steadily increasing ( Figure 3). While only one scientist was contributing in November 2017, 23 scientists from six institutions (University of Toronto, University of Oxford, University of North Carolina, University of Leicester, the Karolinska Institute in Sweden and University of Montpellier in France) are recording their work at openlabnotebooks.org as of December 2018.

As importantly, impact is also increasing, judging by the average number of views per experimental record calculated from statistical data available at Zenodo.org ( Figure 3). Some reports raised a considerable interest. For instance, the crystal structure of USP5 in complex with small molecule fragments has 821 unique views and 324 unique downloads as of December 2018 ^17^. If the initiative is successful, we anticipate that within three to five years, usage metrics are comparable at openlabnotebooks.org and bioRxiv, the preprint server for biology.

Data posted at openlabnotebooks.org are raising interest in academic groups, but also in the industry. For instance, a notebook contributor was directly contacted by a big pharmaceutical company to further discuss the results that he had shared online, and a big biotech company asked permission to another contributor to include their data in a presentation at a public scientific meeting. Some of the research reported at openlabnotebooks.org is of direct relevance to patient groups. For instance, four scientists record their results on testing chemical inhibitors of the kinase ALK2, a potential therapeutic target for the treatment of the pediatric brain tumor diffuse intrinsic pontine glioma (DIPG), and the heterotopic ossification disorder fibrodysplasia ossificans progressive (FOP) ^18, 19^. The compounds, developed by the open science biotech company M4KPharma, are still in pre-clinical phase of development but should ultimately lead to clinical trials for these incurable diseases ^20^. Scientists working on projects with a clear path to the clinic are eager to share their enthusiasm and commitment with patient groups (sometimes using social media to announce their latest open notebook post) who, in turn, follow their work.

## The challenges of open lab notebooks

Three antagonizing points that inhibit scientists from starting their own open lab notebook are the fear of being scooped, the inability to report collaborative work when collaborators want to keep data secret, and the concern that an open notebook will take time away from an already overburdened schedule ^21^. The language barrier for non-native English speakers, and the availability of open lab notebook solutions can also be challenging. It is indeed likely that maintaining an open lab notebook increases the chances of being scooped, but it is too early at this point to know whether this effect is minor or significant. Paradoxically, and given the territorial nature of the current frameworks for funding and managing scientific research, entries in one’s open lab notebook may mark one’s area very effectively, especially in a conceivable future when funding trusts and councils start looking into them. We would argue that most, if not all, scientists get scooped during their career, and that open lab notebooks serve as a safety net for early career scientists who have a citable record of their work if they ever get scooped. Obtaining permission from collaborators to report collaborative work in open lab notebooks can be challenging. We believe that the best way to avoid such a situation is to clearly state at the outset of a collaboration the intention to adopt open science principles ^22^. Scientists are more likely to agree if presented with the idea well in advance. The time invested in practicing clear, concise and engaging scientific writing is not lost on one’s career. After some practice, maintaining an open lab notebook should not take more time than using a regular lab notebook.

Open notebooks being published before peer-review, there is a risk that dubious experiments, erroneous analysis or misinterpretations find their way on open platforms, get amplified over the internet and mislead colleague scientists, patient groups or other communities. Once they become indexed by popular search engines, open lab notebooks could become a source of pollution of the scientific (and non-scientific) literature. This risk, which is not limited to open notebooks but extends to the increasing number of Journals that adopt a post-publication peer-review mechanism, is real, serious, and should be monitored. We believe that the best way to mitigate this risk is for open notebooks to provide a platform for open comments. In principle, this could be an even stronger quality control than the current peer-review system in place in most scientific journals, as the number of “open reviewers” for any given report is limitless. At the moment, we find that very few comments are posted at openlabnotebooks.org, a platform that is only a year old, but we see that comments are mainstream, and sometimes turn into healthy discussions at Open Source Malaria, a pioneer in the field ^23^.

## Future directions and conclusion

Open lab notebooks represent a major departure from current practices in science (especially biomedical sciences) and hold a mix of promises and risks. As the community producing these lab notebooks is increasing, there is an opportunity to move beyond ideology and anecdotal data to evidence-based policy design. In the spirit of openness, we call on colleagues from both the life science and the social sciences communities to conduct systematic evaluation of the benefits and downsides of open lab notebooks. It will be important to compare several parameters on a yearly basis. These may include the frequency of research being scooped among scientists disclosing their work in open lab notebooks versus a less open reference group; the frequency of new collaborations; the frequency of comments and ideas received by the authors of open notebooks; and instances where open lab notebooks were essential for compliance with funder or institutional requirements. More difficult to assess will be issues such as recognition, career progression, speeding up research, and impact on reproducibility, but they could all be addressed with appropriate questionnaires and data analytics.

Our goal is to see the number of open lab notebooks increase exponentially over the coming years. Future implementation of novel features, such as the ability to search for experiments containing compounds with specific chemical templates, is expected to extend the reach of the platform to medicinal and computational chemists. Indexing of open lab notebooks by popular search engines such as Google Scholar (which already indexes pre-prints and other non-peer-reviewed documents) would increase the visibility and impact of open notebooks. Importantly, open lab notebook data deposited at Zenodo.org is already searchable with Google’s Dataset search engine. To further encourage scientists to break free from the tradition of secrecy that has been passed on for generations, a cultural change needs to be supported at institutional and governmental levels. Funding bodies are starting to define and enforce open science publication practices ^24^. Similarly, universities could take a more proactive role, for instance by including adhesion to open-access principles as an evaluation criteria for career advancement ^25^. Indeed, while strong incentives described above already exist for junior scientists to start their own open lab notebook, the benefit to their PIs who already have established a professional network and don’t need to showcase their skills is not always as clear. As long as scientists are not convinced that open science is good for them, Science 2.0 will have to wait.

## Data availability

No data are associated with this article.
