# Supplementary material for: Open laboratory notebooks: good for science, good for society, good for scientists
Source: F1000Res. 2019 Apr 2;8:87. Originally published 2019 Jan 22. [Version 2] doi: 10.12688/f1000research.17710.2 (PMC6694453; doi:10.12688/f1000research.17710.2)
Supplement: Peer review history

# Peer review: Reviewer response for version 2

**DOI:** 10.5256/f1000research.20551.r50782

**Published:** 2019-08-12

**Authors:** Jean-Baptiste Poline

**Affiliations:** 1 McGill University, McGill University, Montreal, QC, Canada

The article by Matthieu Schapira and Rachel Harding "Open laboratory notebooks: good for science, good for society, good for scientists" is a clear presentation of the benefits of open notebook for the scientific community. The central argument is that one of the main benefits is an increased efficiency (both in time and cost) of the scientific process through the faster re-utilization of positive or negative results. The authors discuss in detail the challenges of the initiative and the results of the current openlabnotebook.org implementation and results.

 I liked in particular the straightforward discussion on the current incentive system from the authors. The perceived or real danger of being scooped is a clear obstacle to the efficient use of these. The arguments of why opennotebooks are good for scientists are clear and compelling although based mostly on anecdotal data.

 The implementation of the infrastructure is solid, reusing standard and long lasting components (zenodo, github). The search capacity across notebooks could have been detailed more. An component around the standardization of terms, use of controlled vocabulary (or even ontologies) could be added in the future. For instance, an extension of the figshare data documentation system would be helpful to label notebook appropriately.

 The uptake in both the building and the use of openlabnotebooks is promising, although absolute number of adaption are still limited since this is still at early stages.  

 The challenges for wider adoption are clear - (namely, fear of being scooped, collaborators with non open practices, and fear that the time invested would not be worth (in terms of recognition), risk of non solid information being released. The solutions to mitigate these challenges are not as well developed, but as this is a new technology and practice, only the future will say if these are indeed going to prevent the openlabnotebooks developments. I think that the close involvement of both national and private funding agencies could rapidly solve for these adoption challenges in a top-down manner.

 Last, I have one question: what is the process to join the openlabnotebooks consortium?

I confirm that I have read this submission and believe that I have an appropriate level of expertise to confirm that it is of an acceptable scientific standard.

# Peer review: Reviewer response for version 2

**DOI:** 10.5256/f1000research.20551.r46587

**Published:** 2019-04-18

**Authors:** Lara M. Mangravite

**Affiliations:** 1 Sage Bionetworks, Seattle, WA, USA

The authors did a reasonable job of addressing my questions.  I have no further concerns.

I confirm that I have read this submission and believe that I have an appropriate level of expertise to confirm that it is of an acceptable scientific standard.

# Peer review: Reviewer response for version 1

**DOI:** 10.5256/f1000research.19363.r43420

**Published:** 2019-03-04

**Authors:** Lara M. Mangravite

**Affiliations:** 1 Sage Bionetworks, Seattle, WA, USA

The opinion piece on Open Laboratory Notebooks written by Dr.s Schapira, Harding, and the Open Lab Notebook Consortium provides a thoughtful commentary on an interesting and relevant topic. The Open Labnotebook provides an interesting and potentially powerful approach to support transition of scientific communication beyond traditional peer-reviewed scholarly publications. As the authors note, the availability of digital media provides a much wider array of approaches to support meaningful research dissemination – and to promote scientific reuse and collaboration.

 The authors speak primarily from their own experience – and, because the use of open lab notebooks is quite new, there is often not a lot of data to back up their comments. As such, it was hard to understand what ideas were well supported and what ideas were in need of evaluation. Along those lines, the authors do not put their work into context of the broader field – I do not have a clear sense of who else is using an open labnotebook approach, whether their experiences are similar or different, and what portions of the scientific community were finding open labnotebooks useful. This context would help in synthesizing the ideas in the manuscript.  

 Secondly, it would be enormously helpful to understand how the authors anticipate that open labnotebooks would operate in the context of the current scientific communication framework. Do you expect labnotebooks to replace peer-review journals or to complement them? How might a researcher convey the overarching hypothesis that they are looking to address through the set of experiments run across a series of notebook entries? It seems that neither the labnotebook approach nor the existing publication approach are sufficient to fully convey scientific output by themselves. I would very much enjoy understanding the authors’ perspective on this.

 Along these same lines, I am curious as to how notebook readers will incorporate this form of communication into their workflow. It will have to be useful – and easy – in order to gain the desired level of adoption. I appreciated the inclusion of the notebook use statistics – it would be helpful to understand how users found these posts, what kind of users were attracted, and how they responded to this medium as a form of scientific discourse. 

 The role of the gatekeeper was also not addressed. Open LabNotebooks are reliant on a series of technical platforms. These platforms will have an impact on the overall success based on their sustainability models, their archiving approaches, and their need for financial viability. Is there a cost to using the system – and, if not, how is it funded? Are the content fully owned by the authors and do they remain under full control of the authors? What are the responsibilities of the platforms and how might their choices impact the approach? 

 Finally, the authors have a tremendous opportunity to ground some of this theoretical work in their own practical application. It would be useful to understand how your scientists’ experiences compared to those were anticipated. For example, one frequent concern heard by researchers is one of time – they are worried that use of the system will require an additional burden in an already over-committed schedule. How much time do the current users spend on their posts? Did this reduce over time? This same approach can be used to address the anticipate benefits. 

I confirm that I have read this submission and believe that I have an appropriate level of expertise to confirm that it is of an acceptable scientific standard, however I have significant reservations, as outlined above.

# Response

**Published:** 2019-03-26

**Authors:** Matthieu Schapira

*The authors speak primarily from their own experience – and, because the use of open lab notebooks is quite new, there is often not a lot of data to back up their comments. As such, it was hard to understand what ideas were well supported and what ideas were in need of evaluation. Along those lines, the authors do not put their work into context of the broader field – I do not have a clear sense of who else is using an open labnotebook approach, whether their experiences are similar or different, and what portions of the scientific community were finding open labnotebooks useful. This context would help in synthesizing the ideas in the manuscript.*

 Open lab notebooks remain a niche of scholarly communication and the number of active examples is limited. An additional section has been added in “Implementation of an open lab notebook platform” describing the brief history and status of the open lab notebook community:

 “Open lab notebooks have been pioneered and championed by a number of practitioners but remain a niche activity in the scientific community. Jean-Claude Bradley first coined the term “open notebook science” in 2006 and his definition of this method of scholarly communication have laid the foundations for our own efforts [12]. In addition to the notebooks of individual researchers following Bradley’s template, open notebook examples now include collective efforts from the Open Lab Notebook Network (http://onsnetwork.org) and Open Source Malaria (http://opensourcemalaria.org). However, the open lab notebook community remains small, the practice is not consistently defined or implemented and the impact of these efforts in the field have not been systematically evaluated.”

*Secondly, it would be enormously helpful to understand how the authors anticipate that open labnotebooks would operate in the context of the current scientific communication framework. Do you expect labnotebooks to replace peer-review journals or to complement them? How might a researcher convey the overarching hypothesis that they are looking to address through the set of experiments run across a series of notebook entries? It seems that neither the labnotebook approach nor the existing publication approach are sufficient to fully convey scientific output by themselves. I would very much enjoy understanding the authors’ perspective on this.*

 We see open lab notebooks as a mechanism for scientists to share their work before they publish it in peer-reviewed journals. As such, they are complementary to peer-reviewed publication. This is specified in the article as follows:

 “We believe that open laboratory notebooks, where research scientists record their work online and in near-real time, are an efficient way to disseminate data before it is published in peer-reviewed journals, and …”

*Along these same lines, I am curious as to how notebook readers will incorporate this form of communication into their workflow. It will have to be useful – and easy – in order to gain the desired level of adoption. I appreciated the inclusion of the notebook use statistics – it would be helpful to understand how users found these posts, what kind of users were attracted, and how they responded to this medium as a form of scientific discourse.*

 We agree that much is to be gained from knowing the demographics and motivations of open lab notebook readers. The readers input in the well populated comment section of the platform Open Source Malaria strongly suggest that most readers are scientists in the field, sharing common goals and interests.

*The role of the gatekeeper was also not addressed. Open LabNotebooks are reliant on a series of technical platforms. These platforms will have an impact on the overall success based on their sustainability models, their archiving approaches, and their need for financial viability. Is there a cost to using the system – and, if not, how is it funded? Are the content fully owned by the authors and do they remain under full control of the authors? What are the responsibilities of the platforms and how might their choices impact the approach?*

 The server hosting openlabnotebooks.org is maintained by the Structural Genomics Consortium at University of Oxford. It relies on Wordpress, which is an open source platform, and requires limited maintenance. Consequently, the service is free for both contributors and readers. Zenodo is hosted by the CERN and is also free. The issue of permanence, sustainability, archiving and ownership are important ones and are addressed in our response to Reviewer 1 (sections #6 and #17) and discussed in the revised version of the manuscript.

*Finally, the authors have a tremendous opportunity to ground some of this theoretical work in their own practical application. It would be useful to understand how your scientists’ experiences compared to those were anticipated. For example, one frequent concern heard by researchers is one of time – they are worried that use of the system will require an additional burden in an already over-committed schedule. How much time do the current users spend on their posts? Did this reduce over time? This same approach can be used to address the anticipate benefits.*

 When initiating this project, prospective open notebookers were surveyed regarding their concerns, questions and attitudes about running their own open notebook projects which highlighted many of the concerns you raise, in particular, the time burden. We are planning to conduct a follow-up survey once the number of scientists joining our platform increases.

# Peer review: Reviewer response for version 1

**DOI:** 10.5256/f1000research.19363.r43417

**Published:** 2019-02-06

**Authors:** Matthew H. Todd, Edwin Tse, Marat Korsik, Mathamsanqa Bhebhe

**Affiliations:** 1 School of Pharmacy, University College London (UCL), London, UK; 2 University of Sydney, Sydney, Australia

This opinion piece is on a timely, important topic and is clearly and engagingly written. Anecdotally, we find that many of our colleagues in science are unaware that open lab notebooks exist. This article will help.

 The authors identify several important advantages and challenges associated with the near-immediate deposition of results into the public domain, online. They use examples from their own research to highlight the possibilities.

 The refereeing team behind this review are seasoned users of open lab notebooks, and so are in a good position to judge the piece. We judge it to have cleared peer review from our perspective, once the following comments and suggestions have been acted upon. There are a number, which should be read not as criticism but as testament to our shared enthusiasm for this subject and its importance in the future of research.

 1) **Secrecy**. In the introduction, reasons are suggested for why scientists may keep results secret. We would suggest that there are two important reasons that are not explicitly mentioned: i) that the scientist may want to patent something, and ii) that the scientist cannot be bothered to work out how to release research using atypical means. The first point is alluded to where mention is made of ownership, and the second point is alluded to by the mention of "paper" but we would argue these two factors are significant enough that they should be made explicit.

 2) **Careers**. We'd be interested in whether there is a justification for the statement "Many believe that openly sharing work online will limit career opportunities." If there is none, then perhaps rephrase this more as a possibility?

 3) **Grants**. The statement "Grant applications that highlight the use of open lab notebooks are being viewed positively" may be true (one hopes it is), but the evidence presented doesn't support that statement (the grants may have been funded because the science was so good, regardless of the dissemination plan), so again, this probably needs to be made more aspirational.

 4) **Errors**. The authors need to address what happens if an experiment is recorded containing a mistake, and a mistake that might propagate *via* an ungrounded conclusion. Is there a danger in leading colleagues down the wrong path? Is there a danger to the reputation of young scientists? Would incorrect conclusions, if indexed by search engines, lead to literature pollution that might be hard to correct?

 5) **Machines**. One must make the assumption that few people will ever *read* open lab notebooks, just as few people read regular lab notebooks. More needs to be made of this, since a) it is essential, rather than just desirable, that the notebook is searched and indexed by e.g. Google, and b) the entries need, ideally, to be machine readable. Could the authors comment on this - i.e. how Googleable the contents are (beyond the Dataset Search they mention) and what can be done to ensure that the entries can be understood by machines?

 6) **Permanence**. There are many web links in the article. This is an academic publication, which is intended to last forever. It's likely those URLs will not last forever. Is there a way that the pages pointed to can be archived somehow? An important, relevant example: this manuscript does not refer to one of the pioneers of open lab notebooks - indeed he coined the phrase "open notebook science" - Jean-Claude Bradley. Bradley's blog can still be accessed but the wiki that used to house all the raw data has gone. In publishing our most recent paper that made use of open lab notebooks ^1^, we took pains to archive the lab notebooks on a repository to mitigate potential loss by external providers, and to back up web pages (as PDFs) to similar places. Can the authors address the two issues here: i) should we link to web pages in academic articles without backing them up? 2) How are the lab notebooks backed up and archived?

 7) **Time Stamps**. The authors mention "results in open lab notebook are date-stamped." It would be interesting to know whether the authors have looked into whether the date stamps are "convincing" from a legal standpoint. Would they, for example be sufficient to claim priority in a legal challenge? Is there an issue with the fact that ELN pages can be edited, over an extended period of time, or is it sufficient to ensure that the page has a robust revision history?

 8) **Scooping**. A scooper could claim that they were simply unaware of the open scientist's work. This seems a reasonable defense. What needs to happen for ignorance of an open lab notebook not to be a defense against ignorance of prior art?

 9) **Citations**. We are cited twice (thank you), but there are two other relevant papers the authors might be interested in citing should these be deemed by them to be appropriate: i) an extensive discussion on the use cases of an open source ELN called Labtrove ^2^ pioneered by Jeremy Frey's team at Southampton, and ii) a large medchem project by Open Source Malaria which was conducted entirely using open lab notebooks ^1^ and which may be instructive in terms of how to publish a paper based on open notebook work (also described informally here.

 10) **Examples of Inputs**. Are there any examples the authors can point to where the open ELN has helped the research, e.g. where suggestions have been made, and acted upon, to help the science?

 11) **Examples of ELNs**. Are there other examples of open lab notebooks being used in biomedical research? Is this, in fact, still a highly niche activity?

 12) **Language**. The language barrier is mentioned, but we're not sure this is relevant to this paper. Language is already a barrier in the current system, though English is essentially a *lingua franca* across science.

 13) **Licence**. What is the license that covers the authors' i) blog posts and ii) notebooks? To what extent is this an important choice? For example, can others take and re-use the content without restriction, or only for non-commercial purposes?

 14) **Raw Data**. The authors mention that the ELN entries include "all data". This is an important feature of open ELNs that distinguishes the practice from a great deal of open science in which highlights might be discussed, or blogged about, but without the attendant raw data. We investigated briefly to see how much of the raw data could be accessed in the authors' cases. We have noticed that a fair number of the lab notebook entries recorded by the team in Zenodo contains PDF/Word-style summaries of data, rather than the data themselves. This limits re-use of the data. Could the authors comment on the central importance of the availability of all raw data?

 Examples:

- This blog post mentions performing synthesis of analogues based on lit procedures but there’s no link to an ELN.
- Same with this page.
- And this.
- This page has a link (in the 2nd paragraph) to a (structure?) data file but no backlink from the data back to the blog page, risking orphaning of content?
- This blog page does have a link to the ELN. There is more experimental detail, but there is no raw characterization data. Also the backlink to the blog page is broken.
- Many biology entries contain pictures of purifications and for the most part, the ELN pages only have a docx or pdf there. Should there be any raw data available for these kinds of pages, or are docx and PDF enough?

15) **Identifiers**. It is mentioned that is is desirable to have "the ability to search for experiments containing compounds with specific chemical templates". We agree. Some ELNs already allow this (e.g. Chemotion ^3^ and C6H6 ^4^). We have found a stop-gap is the manual inclusion of chemical strings such as SMILES, InChI. Can the authors comment on whether similar things can be included in order to allow machines to understand the biology contained within the entries, e.g. UniProt numbers?

 16) **Metrics *re* Access**. Is there a way of distilling out access by non-team people, or even access by the author of the page who may have accessed multiple times during the editing of a page?

 17) **Ownership**. When a researcher moves on, does that researcher have any responsibility towards the ELN? e.g. if a mistake is found, who needs to correct it? Is it the responsibility of the PI to act as future curator?

We confirm that we have read this submission and believe that we have an appropriate level of expertise to confirm that it is of an acceptable scientific standard, however we have significant reservations, as outlined above.

## References

[1] : Open Source Drug Discovery: Highly Potent Antimalarial Compounds Derived from the Tres Cantos Arylpyrroles. *ACS Cent Sci*.2016;2(10) : 10.1021/acscentsci.6b00086 687-701 10.1021/acscentsci.6b00086 27800551PMC5084075

[2] : Experiences with a researcher-centric ELN. *Chem Sci*.2015;6(3) : 10.1039/c4sc02128b 1614-1629 10.1039/c4sc02128b 29308130PMC5639792

[3] : Chemotion ELN: an Open Source electronic lab notebook for chemists in academia. *J Cheminform*.2017;9(1) : 10.1186/s13321-017-0240-0 54 10.1186/s13321-017-0240-0 29086216PMC5612905

[4] : The C6H6 NMR repository: An integral solution to control the flow of your data from the magnet to the public. *Magn Reson Chem*.2018;56(6) : 10.1002/mrc.4669 520-528 10.1002/mrc.4669 28981966

# Response

**Published:** 2019-03-26

**Authors:** Matthieu Schapira

*1) **Secrecy**. In the introduction, reasons are suggested for why scientists may keep results secret. We would suggest that there are two important reasons that are not explicitly mentioned: i) that the scientist may want to patent something, and ii) that the scientist cannot be bothered to work out how to release research using atypical means. The first point is alluded to where mention is made of ownership, and the second point is alluded to by the mention of "paper" but we would argue these two factors are significant enough that they should be made explicit.*

 Points well taken. The following statement was added to the Introduction “…and can be compounded by constraints associated with patent protection procedures or the absence of clear mechanism to make one’s data publicly available.”

*2) **Careers**. We'd be interested in whether there is a justification for the statement "Many believe that openly sharing work online will limit career opportunities." If there is none, then perhaps rephrase this more as a possibility?*

 This was not clear. The sentence was replaced as follows:

 “Many believe that the chances of getting scooped before one publishes their work in a peer-reviewed journal increase when openly sharing their work online [9]”

*3) **Grants**. The statement "Grant applications that highlight the use of open lab notebooks are being viewed positively" may be true (one hopes it is), but the evidence presented doesn't support that statement (the grants may have been funded because the science was so good, regardless of the dissemination plan), so again, this probably needs to be made more aspirational.*

 This was revised as follows:

 “Our personal observations seem to indicate that grant applications highlighting the use of open lab notebooks are being viewed positively.”

*4) Errors. The authors need to address what happens if an experiment is recorded containing a mistake, and a mistake that might propagate via an ungrounded conclusion. Is there a danger in leading colleagues down the wrong path? Is there a danger to the reputation of young scientists? Would incorrect conclusions, if indexed by search engines, lead to literature pollution that might be hard to correct?*

 We agree that this important point was missing. The following was added to the section “The challenges of open lab notebooks”:

 Open notebooks being published before peer-review, there is a risk that dubious experiments, erroneous analysis or misinterpretations find their way on open platforms, get amplified over the internet and mislead colleague scientists, patient groups or other communities. Once they become indexed by popular search engines, open lab notebooks could become a source of pollution of the scientific (and non-scientific) literature. This risk, which is not limited to open notebooks but extends to the increasing number of Journals that adopt a post-publication peer-review mechanism, is real and serious. We believe that the best way to mitigate this risk is for open notebooks to provide a platform for open comments. In principle, this could be an even stronger quality control than the current peer-review system in place in most scientific journals, as the number of “open reviewers” for any given report is limitless. At the moment, we find that very few comments are posted at openlabnotebooks.org, a platform that is only a year old, but we see that comments are mainstream, and sometimes turn into healthy discussions at Open Source Malaria, a pioneer in the field.

*5) **Machines**. One must make the assumption that few people will ever read open lab notebooks, just as few people read regular lab notebooks. More needs to be made of this, since a) it is essential, rather than just desirable, that the notebook is searched and indexed by e.g. Google, and b) the entries need, ideally, to be machine readable. Could the authors comment on this - i.e. how Googleable the contents are (beyond the Dataset Search they mention) and what can be done to ensure that the entries can be understood by machines?*

 We believe that in the life sciences, most would find the constraint of formatting their notebook in a standardized language an unacceptable burden. Open lab notebooks should be indexable by Google or other search engines as free text records. Chemists would benefit from the integration of cheminformatics functionality, such as fingerprint-based substructure or similarity searches. These tools are openly provided by RDKit, and we expect that they will soon be integrated into open lab notebook platforms.

*6) **Permanence**. There are many web links in the article. This is an academic publication, which is intended to last forever. It's likely those URLs will not last forever. Is there a way that the pages pointed to can be archived somehow? An important, relevant example: this manuscript does not refer to one of the pioneers of open lab notebooks - indeed he coined the phrase "open notebook science" - Jean-Claude Bradley. Bradley's blog can still be accessed but the wiki that used to house all the raw data has gone. In publishing our most recent paper that made use of open lab notebooks ^1^, we took pains to archive the lab notebooks on a repository to mitigate potential loss by external providers, and to back up web pages (as PDFs) to similar places. Can the authors address the two issues here: i) should we link to web pages in academic articles without backing them up? 2) How are the lab notebooks backed up and archived?*

 We appreciate this concern. We have multiple layers of backup for the openlabnotebooks.org web-site including snapshots, replication, and off-site tape storage.   We also export all blog posts automatically at the end of each week to the following GitHub repository https://github.com/thesgc/static-openlabnotebooks. The Information Technology Services at University of Toronto is now also maintaining an archive of the website at https://wayback.archive-it.org/6473/*/https:/opennotebook.thesgc.org/, which is updated quarterly. The data posted on Zenodo is also backed-up, as specified at https://about.zenodo.org/infrastructure/. Archiving each website that one cites is an impressive commitment to the permanence of one’s work. We assume that consent should first be obtained to archive someone else’s research output. We are not planning to follow this path at the moment. The following statement was added to the article:

 “The blogs, posted at openlabnotebooks.org, are managed by a webserver downloaded from wordpress.org, archived weekly to GitHub (repository https://github.com/thesgc/static-openlabnotebooks), quarterly to archive.org (https://wayback.archive-it.org/6473/*/https:/opennotebook.thesgc.org/), and link…”

 We also added the following section:

 “Open laboratory notebooks need to guarantee that the data will remain accessible, in order to avoid the fate suffered by the pioneer open notebook of Jean-Claude Bradley, which is still accessible while its associated raw data wiki is not. Zenodo is strongly committed to preserving the data it archives. CERN has existed since 1954 and has an experimental program defined for the next 20+ years. Each file copy has two replicas located on different disk servers. In the highly unlikely event that Zenodo closes operations, they guarantee migration of all content to other suitable repositories, and since all uploads have DOIs, citations and links to Zenodo resources (including data) will not be affected.”

*7) **Time Stamps**. The authors mention "results in open lab notebook are date-stamped." It would be interesting to know whether the authors have looked into whether the date stamps are "convincing" from a legal standpoint. Would they, for example be sufficient to claim priority in a legal challenge? Is there an issue with the fact that ELN pages can be edited, over an extended period of time, or is it sufficient to ensure that the page has a robust revision history?*

 We believe that whether a particular electronic date stamp is sufficient to prove the date of disclosure would depend on how well the system was configured and how good the records were logged. Website businesses do depend on date stamping to establish when a particular change to their terms & conditions was made, for example. Our understanding is that the system implemented at Zenodo clearly indicates when a record was posted and when it was edited, which should be sufficient evidence for open notebooks to count as prior art.

 To clarify the date-stamp/versioning mechanism in place at Zenodo, we added the statement “once a record has been published, it can no longer be modified, but revised versions can be appended if necessary”

*8) **Scooping**. A scooper could claim that they were simply unaware of the open scientist's work. This seems a reasonable defense. What needs to happen for ignorance of an open lab notebook not to be a defense against ignorance of prior art?*

 We believe that scientists learn to live with the potential and reality of being scooped. Plagiarism is another problem, but we believe that it is more an academic conduct issue than a legal issue. If materials & methods and results published in a date-stamped open notebook are later authored by another scientist in a peer-reviewed journal, this should in principle raise reputational problem for that person.

*9) **Citations**. We are cited twice (thank you), but there are two other relevant papers the authors might be interested in citing should these be deemed by them to be appropriate: i) an extensive discussion on the use cases of an open source ELN called Labtrove ^2^ pioneered by Jeremy Frey's team at Southampton, and ii) a large medchem project by Open Source Malaria which was conducted entirely using open lab notebooks ^1^ and which may be instructive in terms of how to publish a paper based on open notebook work (also described informally here.*

 We added a citation to the LabTrove paper in the section “Implementation of an open lab notebook platform”, and to the Open Source Malaria paper just above the “Future directions and conclusion” section.

*10) **Examples of Inputs**. Are there any examples the authors can point to where the open ELN has helped the research, e.g. where suggestions have been made, and acted upon, to help the science?*

 Yes. Examples include researchers from a pharmaceutical company that contacted a scientist to further discuss his results, a big biotech company that kindly asked permission to include data from an open notebook at a conference talk, and academics interested in specific chemical templates included in an open report.

 Rachel Harding’s open notebook has had input from a number of readers including Professor Ray Truant (McMaster University) and Professor Jeff Carroll (Western Washington University), both of whom have initiated discussions about the research questions Dr. Harding is pursuing and ultimately lead to collaborative experimental design and analysis of data.

*11) **Examples of ELNs**. Are there other examples of open lab notebooks being used in biomedical research? Is this, in fact, still a highly niche activity?*

 Open lab notebooks are far from mainstream and the number of active examples remains small. However, we enjoy following contributors of the Open Notebook Science Network http://onsnetwork.org/ amongst others.

*12) **Language**. The language barrier is mentioned, but we're not sure this is relevant to this paper. Language is already a barrier in the current system, though English is essentially a lingua franca across science.*

 We agree, but we have examples of scientists who do not feel confident enough in English to use our platform.

*13) **Licence**. What is the license that covers the authors' i) blog posts and ii) notebooks? To what extent is this an important choice? For example, can others take and re-use the content without restriction, or only for non-commercial purposes?*

 We currently license all of our open notebook blog posts and Zenodo deposits using a Creative Commons Attribution license. This allows our work to be shared and reused as widely as possible whilst asking for attribution of the original work only. More complex licenses may put off readers of the notebooks from reusing the content, which is in opposition of the open lab notebook ethos.

*14) **Raw Data**. The authors mention that the ELN entries include "all data". This is an important feature of open ELNs that distinguishes the practice from a great deal of open science in which highlights might be discussed, or blogged about, but without the attendant raw data. We investigated briefly to see how much of the raw data could be accessed in the authors' cases. We have noticed that a fair number of the lab notebook entries recorded by the team in Zenodo contains PDF/Word-style summaries of data, rather than the data themselves. This limits re-use of the data. Could the authors comment on the central importance of the availability of all raw data?*

**

*Examples:*

- *This blog post mentions performing synthesis of analogues based on lit procedures but there’s no link to an ELN.*
- *Same with this page.*
- *And this.*
- *This page has a link (in the 2nd paragraph) to a (structure?) data file but no backlink from the data back to the blog page, risking orphaning of content?*
- *This blog page does have a link to the ELN. There is more experimental detail, but there is no raw characterization data. Also the backlink to the blog page is broken.*
- *Many biology entries contain pictures of purifications and for the most part, the ELN pages only have a docx or pdf there. Should there be any raw data available for these kinds of pages, or are docx and PDF enough?*

We agree that all raw data should be included in open notebook reports, and we encourage scientists using openlabnotebooks.org to adhere to this principle. We are also aware that enforcing virtuous habits can put-off new users, and are opting for a more permissive approach, where we prefer to educate in best practices rather than imposing them.

*15) **Identifiers**. It is mentioned that is desirable to have "the ability to search for experiments containing compounds with specific chemical templates". We agree. Some ELNs already allow this (e.g. Chemotion ^3^ and C6H6 ^4^). We have found a stop-gap is the manual inclusion of chemical strings such as SMILES, InChI. Can the authors comment on whether similar things can be included in order to allow machines to understand the biology contained within the entries, e.g. UniProt numbers?*

 Algorithm could easily be integrated in an notebook platform to interpret UniProt numbers. For machine to understand the biology associated with a UniProt number, if there is such a thing, is beyond our expertise.

*16) **Metrics re Access**. Is there a way of distilling out access by non-team people, or even access by the author of the page who may have accessed multiple times during the editing of a page?*

 The access metrics are directly generated by Zenodo, provides the number of visits, downloads, unique visits and unique downloads. Even if the author revisits or downloads multiple times his or her record, it would count as a single “unique event”.

*17) **Ownership**. When a researcher moves on, does that researcher have any responsibility towards the ELN? e.g. if a mistake is found, who needs to correct it? Is it the responsibility of the PI to act as future curator?*

 All records posted at openlabnotebooks.org and Zenodo remain indefinitely accessible. Our position is that the labnotebook is owned by the scientist who wrote it.
